# Elucidating the Neural Basis of Bipolar Disorder - Secondary Publication

**DOI:** 10.31662/jmaj.2026-0004

**Published:** 2026-04-17

**Authors:** Tadafumi Kato

**Affiliations:** 1 Department of Psychiatry & Behavioral Science, Juntendo University Graduate School of Medicine, Tokyo, Japan

**Keywords:** bipolar disorder, mitochondria, calcium, paraventricular nucleus of the thalamus, lithium, hyperexcitability, depression

## Abstract

Bipolar disorder is a mental disorder characterized by recurrent episodes of mania/hypomania and depression, with a strong genetic contribution and substantial functional burden. Recent genomic studies implicate multiple risk variants converging on intracellular calcium (Ca^2+^) signaling and synaptic function, while neurons derived from induced pluripotent stem cells of patients with bipolar disorder demonstrate altered neuronal excitability and lithium-responsive phenotypes. Building on early neuroimaging and postmortem observations, accumulating evidence supports the mitochondrial dysfunction hypothesis, which proposes that impaired mitochondrial Ca^2+^ buffering disrupts neuronal Ca^2+^ homeostasis and contributes to mood instability. Diverse findings align with this framework: altered brain energy metabolism, increased mitochondrial DNA (mtDNA) deletions, elevated lactate, reduced mitochondrial gene expression and complex I proteins, enrichment of deleterious de novo and mosaic variants in Ca^2+^ signaling- and mitochondrial/endoplasmic reticulum-related genes, and a higher prevalence of the MELAS (mitochondrial encephalomyopathy, lactic acidosis, and stroke-like episodes)-associated m.3243A>G mutation in individuals with bipolar disorder. Animal models further strengthen causal inference. Neuron-specific *Ant1* knockout mice exhibit reduced mitochondrial Ca^2+^ uptake and serotonergic hyperexcitability, while mice with neuron-specific mutant *Polg* accumulate mtDNA deletions and show recurrent depression-like episodes responsive to lithium and switch-like manic behaviors following treatment with a tricyclic antidepressant, indicating construct, face, and predictive validity. To identify the critical brain substrate, mtDNA deletions were mapped and found to accumulate most prominently in the paraventricular thalamic nucleus (PVT), a serotonergic-recipient hub projecting to limbic circuits involved in emotional salience. Human postmortem single-nucleus analyses reveal marked reductions of PVT neurons and prominent gene expression changes in the PVT, including enrichment of GWAS (genome-wide association study) signals among downregulated genes, as well as neuropathological alterations such as granulovacuolar degeneration in the PVT in late-onset cases. These convergent data suggest that genetically driven Ca^2+^ dysregulation and mitochondrial vulnerability promote circuit-level dysfunction―particularly within the serotonin-PVT-limbic pathway―leading to dysregulated emotion-cognition balance and mood swings.

## What is Bipolar Disorder?

Bipolar disorder is a condition characterized by recurring episodes of mania or hypomania and depression, with a lifetime prevalence of around 1% ^(1)^. It is classified into bipolar I disorder, which involves manic episodes, and bipolar II disorder, which involves hypomanic and depressive episodes. Manic episodes are characterized by elevated mood, grandiosity, reduced need for sleep, talkativeness, and increased activity. Depressive episodes are marked by depressed mood, loss of interest, feelings of guilt, and suicidal ideation. Diagnosis is based on DSM-5 (Diagnostic and Statistical Manual of Mental Disorders, Fifth Edition) criteria after excluding organic mental disorders, endocrinological diseases, and drug-induced mood disorders. Without treatment, recurrence is common, leading to impaired social functioning. Therefore, ongoing treatment combining pharmacotherapy and psychosocial interventions is necessary. Mood stabilizers such as lithium and three antiepileptic drugs (valproic acid, lamotrigine, carbamazepine), as well as atypical antipsychotics, are used ^(2)^. Lithium has established efficacy in preventing recurrence, but its therapeutic range is narrow, and toxic levels are close to the therapeutic range, necessitating regular therapeutic drug monitoring. Effective psychosocial treatments include psychoeducation, interpersonal and social rhythm therapy, family therapy, and cognitive behavioral therapy. It is crucial for patients to understand and accept their illness, maintain a consistent daily rhythm, recognize early warning signs of relapse, and prevent recurrence.

## Etiology

Twin studies of bipolar disorder show a high concordance rate of approximately 80% in monozygotic twins, indicating that genomic factors are involved in its onset. Linkage analysis has failed to identify a single causative gene, while genome-wide association studies have implicated numerous genomic factors involved in intracellular calcium (Ca^2+^) signaling and synaptic function ^(3)^. Studies of rare variants have also shown associations with several genes. Although environmental factors such as perinatal disorders contribute to the onset of bipolar disorder, there is significant overlap with schizophrenia, and no specific environmental factors have been identified.

### Brain

Early postmortem brain studies of individuals with bipolar disorder showed no clear organic changes, but quantitative analyses reported reduced neuronal density in the anterior cingulate cortex, prefrontal cortex, and hippocampus ^(1)^.

Magnetic resonance imaging studies reported early findings such as ventricular enlargement and reduced gray matter volume in the anterior cingulate cortex and insular cortex. Research by an international consortium confirmed widespread cortical thinning, which progresses with disease duration. However, these findings were not observed in patients taking lithium.

#### Studies using patient-derived cells

Research using cells from individuals with bipolar disorder initially focused heavily on blood cells. Elevated baseline intracellular calcium concentrations and enhanced calcium responses to stimulation were reported in platelets and cultured lymphoblasts, considered highly reproducible biological findings. Studies using induced pluripotent stem (iPS) cell-derived neurons have reported abnormalities in differentiation. Additionally, hyperexcitability associated with altered mitochondrial gene expression was observed, and in lithium responders, this improved with lithium treatment ^(4)^.

### Mitochondrial dysfunction hypothesis

In the 1990s, the authors analyzed brain metabolism in individuals with bipolar disorder using ^31^P-MRS (magnetic resonance spectroscopy) and found changes similar to those seen in chronic progressive external ophthalmoplegia (CPEO), a mitochondrial disease. When verifying the accumulation of mitochondrial DNA (mtDNA) deletions, characteristic of CPEO, in postmortem brains of individuals with bipolar disorder, an increase in mtDNA deletions was observed. Based on these findings, we proposed the “mitochondrial dysfunction hypothesis of bipolar disorder” in 2000 ^(5)^. This hypothesis posits that mtDNA mutations impair Ca^2+^ buffering capacity, causing alterations in intracellular Ca^2+^ signaling in neurons, which in turn induce mood fluctuations via impaired neural function.

Subsequent studies in pharmacology, brain imaging, postmortem brain analysis, cell models, and clinical research have shown that lithium regulates mitochondrial function via increased Bcl-2 in the mitochondrial outer membrane; that lactic acid is elevated in the brains of individuals with bipolar disorder; that postmortem brains exhibit reduced expression of mitochondrial-related genes and complex I proteins; that 15%-20% of individuals with mitochondrial disease also have bipolar disorder, that changes in mitochondrial gene expression are seen in patient-derived iPS cells, and that interventions improving mitochondrial function, such as N-acetylcysteine or ketogenic diets, show suggested efficacy. These findings cumulatively support the mitochondrial dysfunction hypothesis in bipolar disorder.

## Genomic Studies

We analyzed sequencing data from trio families (an individual with bipolar disorder and their parents) and searched for de novo mutations. As a result, we found a high frequency of loss-of-function mutations in genes in which such mutations are rarely seen in the general population. The identified de novo mutations were frequently found in genes encoding Ca^2+^ channels and mitochondrial/endoplasmic reticulum proteins.

Furthermore, somatic mutations were frequently observed in genes associated with neurodevelopmental disorders and in the transfer RNA regions of mtDNA. Notably, a significantly higher proportion of individuals with bipolar disorder carried the m.3243A>G mutation, the causative mutation for MELAS, a representative mitochondrial disorder ^(6)^. This result replicates the accumulation of the m.3243A>G mutation in postmortem brain and liver tissues previously reported by the authors and has been confirmed in a recent study employing more rigorous methodology.

### Animal model studies

Mitochondrial diseases are classified into severe, maternally inherited forms caused by heteroplasmic mutations (e.g., MELAS) and adult-onset forms in which nuclear gene mutations secondarily cause mtDNA deletions (e.g., CPEO). The causative genes for the latter include POLG (mtDNA polymerase) and ANT1 (adenine nucleotide translocator 1).

Creation of a mouse with neuron-specific ANT1 knockout revealed reduced mitochondrial Ca^2+^ uptake, enhanced serotonergic activity, and hyperexcitability of serotonergic neurons in the dorsal raphe ^(7)^.

Conversely, mice expressing mutant *Polg* specifically in neurons accumulated mtDNA deletions in the brain, exhibited altered intracellular Ca^2+^ dynamics, and developed recurrent depression-like episodes satisfying DSM-5 criteria for depressive episodes ^(8)^. These episodes were suppressed by lithium, while tricyclic antidepressants induced manic-like behavioral changes. This model was considered to satisfy construct validity, face validity, and predictive validity.

#### Search for the causative brain region

While these results support the mitochondrial dysfunction hypothesis in bipolar disorder, this is not specific to bipolar disorder and is thought to reflect cellular vulnerability. In diabetes, mitochondrial dysfunction in pancreatic β-cells is thought to be involved, and in Parkinson’s disease, mitochondrial dysfunction in dopaminergic neurons of the substantia nigra is thought to be involved. To clarify which brain region or cell type is involved in bipolar disorder, we searched for the region with the highest mtDNA deletion levels in mutant Polg transgenic mice and found that deletions were most accumulated in the paraventricular thalamic nucleus (PVT).

## What Is the PVT?

The PVT receives strong projections from serotonergic neurons and projects to the limbic system. It sends collateral branches to the nucleus accumbens and amygdala, regions involved in contrasting positive and negative emotions. Therefore, it is thought to regulate the intensity (salience) of emotions, independent of their valence (emotional direction). Since both inhibition and activation of the PVT induced depressive-like behavior, this region has been suggested to be a causative brain region in bipolar disorder.

### Human PVT

Research on the human PVT is limited, but calretinin (*Calb2*) is used as a marker. Based on structural connectivity derived from diffusion MRI tractography, a paraventricular region within the medial thalamus showing strong connectivity with the nucleus accumbens and amygdala has been identified. 

Using single-nucleus RNA sequencing (snRNA-seq) of the medial thalamus, we identified a *Calb2*-positive cluster. Spatial transcriptomics revealed that this cell population resides in the paraventricular region and exhibits a gene expression pattern similar to that of the mouse PVT ^(9)^.

## Changes in the PVT in Bipolar Disorder Patients

Single-nucleus RNA sequencing analysis of postmortem thalamus and frontal cortex from individuals with bipolar disorder revealed that the PVT showed the largest change in cell number, with PVT neurons reduced by approximately half ^(9)^. This finding was confirmed by immunostaining with an anti-VGluT2 antibody. Furthermore, when exploring gene expression differences, the PVT showed the greatest differential expression. Genes downregulated in the PVT significantly more frequently exhibited GWAS signals associated with bipolar disorder.

Examination of postmortem brains from individuals with bipolar disorder stored at Tokyo Metropolitan Matsuzawa Hospital revealed granular vacuolar degeneration in five of nine individuals, confirmed by CHMP2B immunostaining ^(10)^. Among the individuals showing granular vacuolar degeneration, four of five had late-onset bipolar disorder.

## Summary

**Figure 1.**
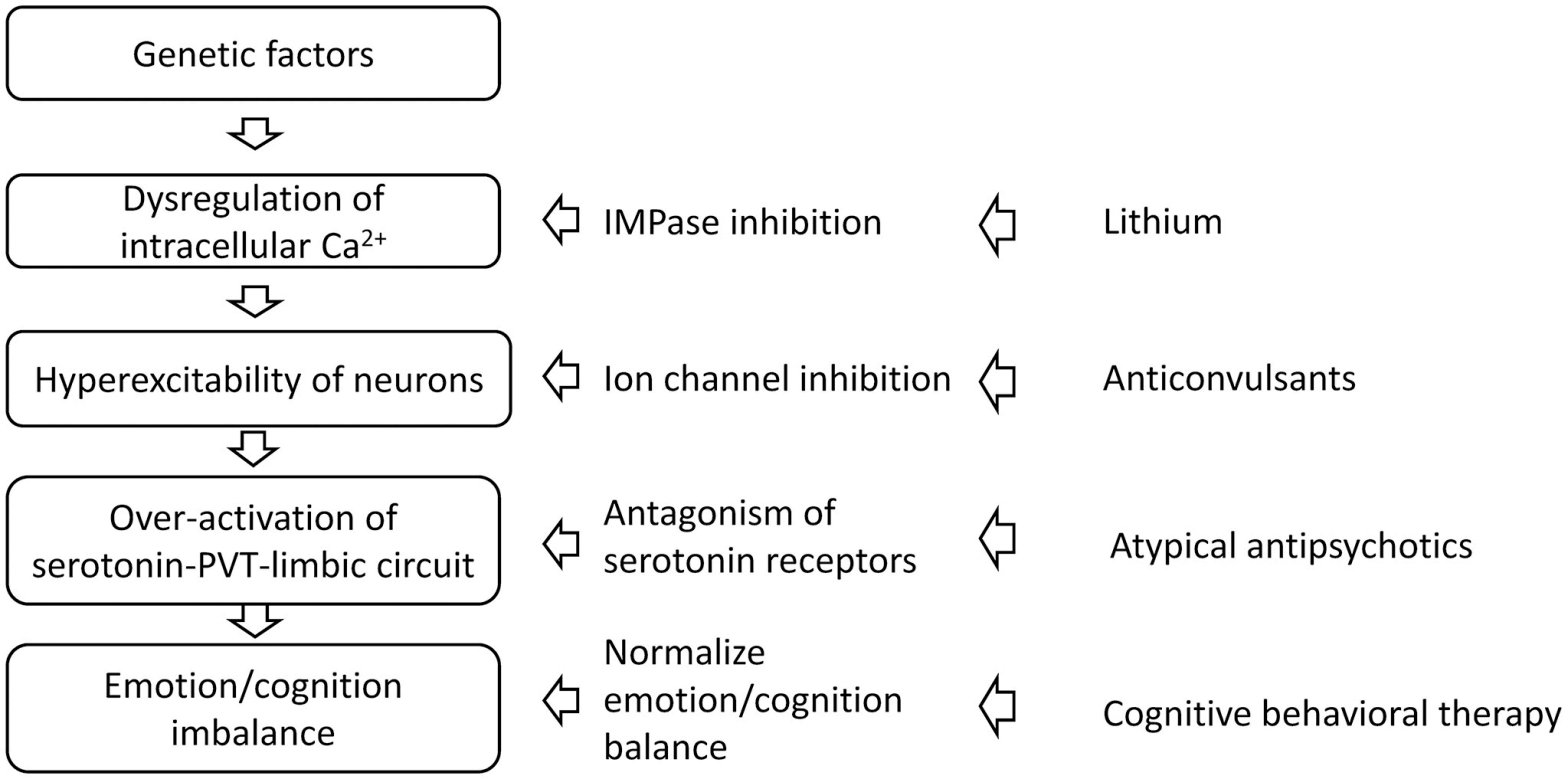
Pathophysiology of bipolar disorder and mechanisms of action of therapeutic agents.

These findings suggest that bipolar disorder arises from genomic factors causing alterations in Ca^2+^ signaling, leading to neuronal hyperexcitability (Figure 1). This triggers overactivity in the serotonin-PVT-limbic circuit, disrupting the balance between emotion and cognition and resulting in mood swings.

As shown in the figure, existing treatments act on different layers of the pathophysiology of bipolar disorder, making their combination likely effective.

Confirmation that the PVT plays a central role in the pathophysiology of bipolar disorder could mark a turning point in bipolar disorder research.

## Article Information

### Acknowledgments

This article is based on the study, which received the Medical Award of the Japan Medical Association in 2025. This is a revised English version of the article originally published in Japanese in the Journal of the Japan Medical Association 2026;154(10):1127～1130 (11). The original version is available at https://med.or.jp/cme/jjma/newmag/pdf/154101127.pdf. Only members of the Japan Medical Association are able to access it.

### Conflicts of Interest

The author received collaborative research funding from Sumitomo Pharma.
